# Evaluation of Circulating Tumor DNA as a Liquid Biomarker in Uveal Melanoma

**DOI:** 10.1167/iovs.65.2.11

**Published:** 2024-02-06

**Authors:** Daniel P. de Bruyn, Natasha M. van Poppelen, Tom Brands, Susanne C. van den Boom, Ellis Eikenboom, Anja Wagner, Monique M. van Veghel-Plandsoen, Geert Geeven, Berna Beverloo, Caroline M. van Rij, Robert M. Verdijk, Nicole C. Naus, Mette M. Bagger, Jens F. Kiilgaard, Annelies de Klein, Erwin Brosens, Emine Kiliç

**Affiliations:** 1 Department of Ophthalmology, Erasmus MC, Rotterdam, The Netherlands; 2 Department of Clinical Genetics, Erasmus MC, Rotterdam, The Netherlands; 3 Erasmus MC Cancer Institute, Erasmus MC, Rotterdam, The Netherlands; 4 Department of Radiation Oncology, Erasmus MC, Rotterdam, The Netherlands; 5 Department of Pathology, Section Ophthalmic Pathology, Erasmus MC, Rotterdam, The Netherlands; 6 Department of Pathology, LUMC, Leiden, The Netherlands; 7 Department of Ophthalmology, Rigshospitalet, Copenhagen University, Copenhagen, Denmark; 8 Department of Clinical Genetics, Rigshospitalet, Copenhagen University, Copenhagen, Denmark

**Keywords:** noninvasive, liquid biopsy, diagnostic, prognostic

## Abstract

**Purpose:**

Uveal melanoma (UM) has a high propensity to metastasize. Prognosis is associated with specific driver mutations and copy number variations (CNVs), but limited primary tumor tissue is available for molecular characterization due to eye-sparing irradiation treatment. This study aimed to assess the rise in circulating tumor DNA (ctDNA) levels in UM and evaluate its efficacy for CNV-profiling of patients with UM.

**Methods:**

In a pilot study, we assessed ctDNA levels in the blood of patients with UM (*n* = 18) at various time points, including the time of diagnosis (*n* = 13), during fractionated stereotactic radiotherapy (fSRT) treatment (*n* = 6), and upon detection of metastatic disease (*n* = 13). Shallow whole-genome sequencing (sWGS) combined with in silico size-selection was used to identify prognostically relevant CNVs in patients with UM (*n* = 26) from peripheral blood retrieved at the time of diagnosis (*n* = 9), during fSRT (*n* = 5), during post-treatment follow-up (*n* = 4), metastasis detection (*n* = 6), and metastasis follow-up (*n* = 4).

**Results:**

A total of 34 patients had blood analyzed for ctDNA detection (*n* = 18) and/or CNV analysis (*n* = 26) at various time points. At the time of diagnosis, 5 of 13 patients (38%) had detectable ctDNA (median = 0 copies/mL). Upon detection of metastatic disease, ctDNA was detected in 10 of 13 patients (77%) and showed increased ctDNA levels (median = 24 copies/mL, *P* < 0.01). Among the six patients analyzed during fSRT, three (50%) patients had detectable ctDNA at baseline and three of six (50%) patients had undetectable levels of ctDNA. During the fSRT regimen, ctDNA levels remained unchanged (*P* > 0.05). The ctDNA fractions were undetectable to low in localized disease, and sWGS did not elucidate chromosome 3 status from blood samples. However, in 7 of 10 (70%) patients with metastases, the detection of chromosome 3 loss corresponded to the high metastatic-risk class.

**Conclusions:**

The rise in ctDNA levels observed in patients with UM harboring metastases suggests its potential utility for CNV profiling. These findings highlight the potential of using ctDNA for metastasis detection and patient inclusion in therapeutic studies targeting metastatic UM.

Currently, the majority of patients with uveal melanoma (UM) are treated with eye-preserving treatments, such as fractionated stereotactic radiotherapy (fSRT), proton beam therapy, and brachytherapy.^1^ These strategies result in retaining the eye and minimizing visual field loss.^2^ The downside is that genetic information of the primary tumor is only to be obtained with a tumor biopsy. Intraocular biopsies could provide this genetic information, but they harbor an inherent risk of (ocular) complications.^3^ They are taken during a trans pars plana vitrectomy that can elevate diagnostic yield, but complications may arise. Persistent hemorrhages,^4^ retinal detachments,^4^ and tumor seeding^5^ have been reported after vitrectomy. On the other hand, fine needle aspiration biopsies are relatively safe, but may have a low diagnostic yield. Tumors with a thickness of less than 5 mm and posteriorly located tumors often have insufficient diagnostic yield.^6^ Endophthalmitis, a rare but severe complication requiring extensive treatment, has been reported after intraocular biopsies.^7^^,^^8^ In most cases, no tissue is obtained for molecular characterization when the diagnosis of the primary tumor is certain. The rationale is that follow-up is similar for all patients regardless of metastatic risk and the potential risk of taking a biopsy. Unfortunately, molecular characterization of tumor tissue is needed for an accurate prediction of prognosis as the prognosis is defined by the presence of mutations in specific driver genes and copy number variations (CNVs). Chromosome 3 loss is associated with the worst prognosis and it co-occurs with a somatic loss-of-function mutation in BRCA1-associated protein 1 (*BAP1*), located on chromosome 3p.^9^^,^^10^ The prognosis of patients harboring a *BAP1*-mutated tumor is worse when tumors acquire additional copies of chromosome 8q.^10^ Disomy 3 tumors mainly comprise tumors with a mutation in X-linked eukaryotic translation initiation factor, 1A (*EIF1AX*) or splicing factor 3B, subunit 1 (*SF3B1*). *SF3B1* mutations occur on hotspot location p.R625 and affect the spliceosome and introduce multiple small chromosomal aberrations. The presence of an *SF3B1* mutation within a tumor results in an intermediate risk of metastases.^11^ Tumors harboring a secondary driver mutation in *EIF1AX* seldom metastasize and generally include a gain of chromosome 6p.^12^^,^^13^ The mutations in primary driver genes are more homogeneous between patients with UMs. Over 90% of the primary driver mutations occur on hotspot locations in 2 genes of the Gαq-pathway: *GNAQ* and *GNA11.*^14^ Less common mutations are reported on hotspot locations in *CYSLTR2* and *PLCB4*. In the majority of studies, a somatic mutation in one of the primary driver genes is identified in all UM tumors.^10^^,^^15^^,^^16^ These somatic mutations do not occur in the germline and are not prognostically relevant,^14^^,^^17^ but are perfectly suited for hotspot mutation detection.

Liquid biopsies could provide information on prognosis and tumor load. One of the liquid biopsies approaches is quantifying and genotyping circulating tumor DNA (ctDNA). The ctDNA are fragments of tumor DNA present in blood and are successfully used as a biomarker in several malignancies. The quantification and CNV-profiling of ctDNA is in the preclinical phase for most malignancies.^18^^–^^20^ In UM, ctDNA is quantified using the hotspot mutations in the primary driver genes.^18^ The levels of ctDNA rise when tumors are larger and metastases arise, which makes it, potentially, a useable tool for follow-up and CNV-profiling.^21^ As ctDNA is shed into the bloodstream when cells go into apoptosis and necrosis,^22^ we expect levels of ctDNA to rise during and after (irradiation) therapy that causes cells to go into apoptosis and necrosis.^23^ These potential causes for an increase in plasma-derived ctDNA prompted us to explore the viability of quantifying and CNV-profiling of ctDNA of patients with UM. In this pilot study, we determined the influence of disease state (i.e. localized and metastatic disease) and eye-sparing fSRT on the amount of ctDNA copies present in plasma and evaluated ctDNA based CNV-profiling of patients with UM prior to treatment, during fSRT, and follow-up of primary and metastatic disease.

## Methods

Thirty-four patients were enrolled for this study of which eight patients were enrolled solely for ctDNA abundancy evaluation (3 patients at diagnosis and during fSRT, 3 patients at diagnosis and metastasis, and 2 patients at the time of metastasis). Ten patients were enrolled for ctDNA abundancy and CNV profiling. Of these patients, ctDNA abundancy was evaluated for two patients at diagnosis and fSRT; one patient at diagnosis, fSRT, and metastasis; four patients at diagnosis and metastasis, and three patients at metastasis. Of these patients, CNV profiling was evaluated for two patients at diagnosis; two patients at fSRT; one patient at localized and fSRT; one patient at the time of diagnosis and metastasis detection; three patients at metastasis detection; and one patient at metastatic follow-up.

**Figure 1.**
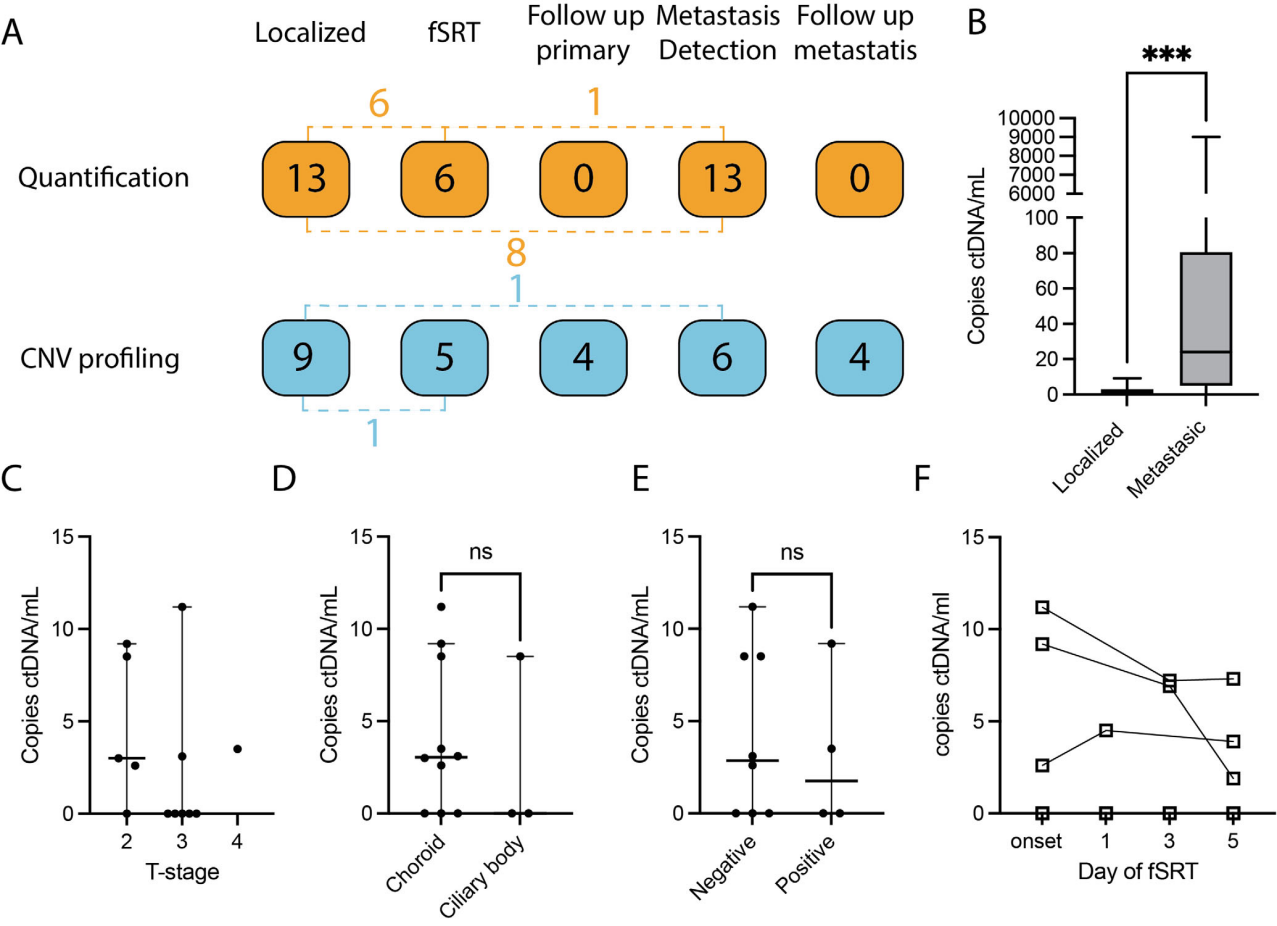
Circulating tumor DNA abundancy and patient inclusion. (**A**) Thirty-four patients were included in this study, the *dashed lines* depict patients that have samples analyzed in different analysis groups (for example, 4 patients had ctDNA quantified at diagnosis and had their ctDNA analyzed for CNV profiling). For ctDNA abundancy, a total of 32 samples from 18 patients were used. Ten samples were used from five patients at localized and fSRT time points. Fourteen samples were used from seven patients at localized and metastatic time points. Three samples were used from one patient at localized, fSRT, and metastatic time points. In the CNV cohort, a total of 28 samples were used from 26 patients. Two samples were used from one patient at fSRT and localized time points. Two samples were used from one patient at localized and metastatic time points (Supplementary Table S1). (**B**) CtDNA was detected in 5 of the 13 patients with localized disease (median = 0 copies/mL) and in 10 of the 13 with metastatic disease (median = 24 copies/mL, ***: *P* < 0.001). (**C**) We compared ctDNA levels of 13 patients in localized disease between tumor characteristics: (**C**) AJCC T-stage, (**D**) tumor location, and (**E**) BAP1 protein expression. The ctDNA abundancy was similar between the tumor characteristics. (**F**) During fSRT, ctDNA abundancy was similar.

Sixteen patients were enrolled for CNV analysis alone (5 patients at diagnosis, 2 patients during fSRT, 4 patients during post-treatment follow-up, 2 patients at metastasis detection, and 3 patients during follow-up of metastases; Fig. 1A, Supplementary Table S1).

For consistency, blood of patients retrieved at the time of diagnosis is referred to as in localized disease in the Results section.

Blood was prospectively collected for CNV analysis and retrospectively for ctDNA abundancy evaluation from 2017 until 2022 and written informed consent was obtained from all patients after ethical approval of the Erasmus MC medical ethics committee (MEC-2009-375). This study adhered to the tenets of the Declaration of Helsinki.

### Blood Collection

Blood was collected in cell-free DNA (cfDNA; Streck, La Vista, NE, USA) and K_2_-EDTA (BD vacutainer systems; BD Plymouth, Plymouth, England, UK) blood collection tubes (BCTs). One ctDNA BCT was used and 2 K_2_-EDTA BCTs, resulting in a maximum total of 32 mL peripheral blood.

Plasma retrieved from EDTA BCTs was isolated by 10 minutes centrifugation at 3500 g at 4°C using a refrigerated centrifuge (Haraeus 5500i; Thermo Fisher, Waltham, MA, USA), followed by 10 minutes of centrifugation at 17.500 g at 4°C using a refrigerated microcentrifuge (Hareaus Fresco 17; Thermo Fisher, Waltham, MA, USA). Plasma retrieved from cfDNA Streck BCTs was isolated by 20 minutes centrifugation at 300 g at room temperature using the refrigerated centrifuge, followed by 19.800g for 10 minutes at 4°C using a cooled centrifuge (Eppendorf centrifuge 5804R, Wesseling, Germany).

Blood was withdrawn within 2 hours after fSRT treatment and subsequently, generally, immediately processed with a maximum of 4 hours after commencing fSRT and 2 hours after blood withdrawal for both cfDNA and K_2_-EDTA BCTs.

### cfDNA Extraction

The cfDNA was isolated from stored plasma using the QIAamp circulating nucleic acid kit (Qiagen, Hilden, Germany) according to the manufacturer's instructions, wherein all buffer and reagent volumes were adjusted to the respective input plasma volumes. The washing column was eluded twice to enhance the cfDNA yield, as reported previously.^24^ Isolated cfDNA samples were subsequently stored at −80°C until further analysis.

### Droplet Digital PCR

For the detection of tumor derived DNA in plasma, previously extracted cfDNA was analyzed using the Stilla Naica crystal digital PCR (dPCR) system (Villejuif, France) and BioRad droplet dPCR system (BioRad, Hercules, CA, USA), according to the manufacturers’ instructions. The Stilla Naica crystal dPCR uses Sapphire consumable chips. The BioRad workflow generates droplets with an Automated Droplet Generator (BioRad), amplifies with a C1000 Touch Thermal Cycler (BioRad), and analyzes with a QX200 system (BioRad). The patients analyzed in this pilot study had known mutations in either in the primary driver genes *GNA11*, *GNAQ*, or *PLCB4*. Subsequently, we used the commercially available BioRAD PrimePCR assays for GNA11 c.626A>T (Gln209Leu), GNAQ c.626A>C (Gln209Pro), and c.626A>T (Gln209Leu), and *PLCB4* c.1888G>T (Asp630Tyr). Mix = 1 µl fluorescein, 5 µl perfecta multiplex master mix, 1 µl mutation and wildtype assay, and 8 µL cfDNA sample was used as the PCR mix and as input for the BioRAD workflow and Stilla Sapphire chips for detection of ctDNA.

The ctDNA fractions were determined using the proprietary Stilla CrystalMiner (version: 2.4.0.3; Villejuif, France) and QuantaSoft (version: 1.7.4; BioRad).

### Shallow Whole Genome Sequencing

The first samples were processed via the NIPT version 1, in which blood was collected in cfDNA BCTs. Blood was centrifuged at 1600 g for 10 minutes at 4°C without brakes, whereafter plasma was aspired. Hereafter, the supernatant was centrifuged at 5600 g for 10 minutes without brakes. The cfDNA was isolated using the QIAsymphony circulating DNA kit (Qiagen), according to the manufacturers’ instructions. The cfDNA is then used for the library preparation and single-end whole-genome sequencing (WGS; 0.2X coverage; 51 bp) using the Illumina HiSeq 4000 or NextSeq 500 (Illumina, San Diego, CA, USA), as previously described.^25^

VeriSeq NIPT solution version 2 (Illumina, San Diego, CA, USA) using the proprietary VeriSeq NIPT Sample Prep Kit (#: 20025895; Illumina, San Diego, CA, USA) was used for blood-based CNV profiling. As per the manufacturers’ instructions, cfDNA was extracted from 1 mL plasma from cfDNA blood collection tubes by adsorption. Library preparation included creating single base overhangs and ligation of indexed adapters, whereafter the cfDNA fragments were purified using solid phase reverse immobilization beads (Illumina, San Diego, CA, USA). Whereafter, individual libraries were quantified before pooling and 36 bp paired-end WGS was performed using a NextSeq 500 and NextSeq 500 reagent kit (Illumina, San Diego, CA, USA) comprising of 20,000,000 to 30,000,000 reads, as previously described.^25^ Bcl2fastq2 (version: 2.20) is used to demultiplex raw sequencing data to create FASTQ files with the automation/demultiplex.sh script. FASTQ files are aligned with BWA mem^26^ creating BAM files. Duplicate reads are marked by picard,^27^ BAM files sorted on coordinates, and SAMtools flagstat output was saved for quality control purposes.

### Clinical Diagnostics and Prognostication

In the clinical diagnostic routing, tumor tissue was used to assess metastatic risk, as previously described.^12^ UM was histologically confirmed and the BAP1 status was evaluated by an ocular oncology pathologist. In short, formalin fixed paraffin embedded (FFPE) slides were made and stained with hematoxylin and eosin to differentiate cell type, evaluate lymphocytic infiltration, mitotic rate, necrosis, and extraocular extension. Periodic acid Schiff staining was used on slides without hematoxylin to evaluate the presence of extracellular matrix loops. BAP1 status was determined by BAP1 staining on FFPE slides, as previously described.^28^ Additionally, single nucleotide polymorphism (SNP)-arrays and a custom IonTorrent panel were used to obtain CNV profiles and detect mutations in driver genes.

CNV profiles were derived from Illumina Q610 SNP-arrays (Illumina, San Diego, CA, USA). DNA was isolated using the QIAamp DNA kit (Qiagen, Hilden, Germany) according to the manufacturer's instructions and 200 ng DNA was used for the Illumina Q610 SNP-array, as previously described.^29^ In short, samples were evaluated using the Quad610 bead-chip, image intensities are extracted using Illumina's Beadscan software and self-normalized in Beadstudio GT (version: 3.0). CNVs were detected by comparing sample data to the Illumina reference set and visualized in Nexus Copy Number (version: 10.1; BioDiscovery, El Segundo, CA, USA). Additionally, a custom panel for the IonTorrent (Thermo Fisher Scientific, USA) platform was developed, as previously described.^29^ In short, DNA was extracted and libraries were constructed with the Ampliseq library kit (Thermo Fisher Scientific, USA) and sequenced using the Ion Genestudio 5 with the custom panel including the primary driver genes *GNAQ*, *GNA11*, *CYSLTR2*, and *PLCB4* and secondary driver genes *MBD4*, *BAP1*, *SF3B1*, and *EIF1AX.*

### Clinical Surveillance for Metastatic Disease

All patients under care in our facility are invited to our outpatient clinic biannually for the first 5 years and every year hereafter, regardless of metastatic risk. During these visits, blood is collected to evaluate the liver function by assessing lactate dehydrogenase (LDH) and gamma-glutamyltransferase (γGT) levels. Additionally, abdominal ultrasounds are used to detect lesions and when lesions are detected computed tomography and magnetic resonance imaging scans are used to confirm metastatic disease.

### Bioinformatic Analyses

Prior studies indicated enhanced detection of ctDNA, when DNA-fragments with a length of 90 to 150 bases were in silico selected.^30^^,^^31^ As UM biomarkers in plasma are scarce, we have enriched for DNA fragment between 80 and 150 bases. Using Samtools,^32^ BAM files of the ShallowSeq samples filtered to only include paired reads with an insert size between 80 and 150 bases.^32^ Hereafter, IchorCNA^33^ was used to estimate the tumor fraction within the total cfDNA fraction, and to detect CNVs originating from the tumor. Next read count files were generated from the BAM files using HMMcopy's readCounter function, as described in the IchorCNA usage instructions. This generated WIG files with 1 Mb bins for chromosomes 1 through 22, X and Y with the recommended minimum mapping quality of 20. With pre-processing finished, the IchorCNA analysis was performed as reported previously, using the R script and reference files included with the tool.^33^ To increase the sensitivity of ctDNA detection, the samples were reprocessed in which the WIG-files were normalized prior to running IchorCNA. The mean read depth of non-zero bases was calculated for chromosome 2q, 4, and 12q, using BEDtools coverage.^34^ These chromosomes were least affected in the total ROMS UM-cohort: chromosomal arm 2q (<1%), chromosome 4 (<5%), and chromosomal arm 12q (<1%) Supplementary Figure S1. This served as the scaling factor applied to the bins. IchorCNA analysis was performed identically to the first. Size selection was not possible for single-end data, but the other procedures were performed in a similar manner. In addition to the IchorCNA CNV detection, Nexus Copy Number 10.0 (BioDiscovery, Hawthorne, CA, USA) was used to detect and visualize CNVs. Nexus BAM MultiReference was used with 2 Mb bin size, 52.5 k target nucleotides per bin for non-size selected samples and 70 k target nucleotides per bin for size selected samples. Reference files were built by using control samples that were analyzed with the same pipeline for both the V1 and V2. Sample reads were rejected when the read depth was <15 and the MAPQ score <30. Probes were recentered around diploid regions, for which chromosome 12q was selected. The significance threshold was set at 10^−8^, a minimum of 3 probes was required and a maximum probe spacing of 1000 kb before cutting the segment was set. The Log ratio for loss and gain were set at −0.015 and 0.035, respectively.

### In Silico Spike In

We have used two control samples (T1 and T2) with a diploid genome and similar total read counts and insert size patterns, as determined by SAMtools flagstat.^32^

To create a gain of chromosome 8q, we have used T2 to extract the reads on chromosome 8q (excluding 1 Mb from the centromere) with SAMtools view -b.^32^ Afterward, subsamples were made of the paired and shuffled reads with fractions of 0.1%, 0.2%, 0.5%, 1%, 5%, 10%, and 25% using the SAMtools view -b -s INT.FRAC function.^32^ The subsamples were then merged with the T1 control sample SAMtools.^32^

For the loss of chromosome 3, we have created a BAM file with a total loss of chromosome 3 from the FASTQ file of T1. Chromosome 3 was removed from the reference genome. BWA index was used to index this file and BWA mem was used create a new BAM file.^26^ Afterward, reads of chromosome 3 were extracted from the original T1 sample and merged with the edited sample in a similar manner as the chromosome 8q gain, but with fractions of 99.9%, 99.8%, 99.5%, 99%, 95%, 90%, and 75%. The samples with chromosome 3 loss and chromosome 8q gain were then merged and indexed with SAMtools^32^ for the 7 respective percentages of loss and gain (i.e. 0.1%, 0.2%, 0.5%, 1%, 5%, 10%, and 25% tumor load).

### Statistical Analyses

Statistical analyses were conducted with Graphpad Prism (version 9.4.1) software (Graphpad software LLC, Boston, MA, USA). The *P* values were calculated with the ANOVA and *t*-test, and Kruskal-Wallis and Mann-Whitney *U* test for Gaussian and non-Gaussian distributed continuous variables, respectively, and Fisher's exact test and Chi-square test for categorical values. All statistical tests were two-sided, and statistical significance was defined as a *P* value of less than 0.05.

## Results

Blood was prospectively collected for tumor load evaluation and CNV-profiling between April 2017 and June 2022 next to routine diagnostics (graphical abstract). In this pilot study, a total of 34 patients were included (see Fig. 1A), consisting of 22 female and 12 male patients. The median age at onset for this cohort was 63.6 years (range = 75 years). Molecular profiling of the tumor was performed for 22 of 34 patients, of which 18 patients had a BAP1-negative tumor and 4 patients had a BAP1-positive tumor. The median follow-up of patients was 28.2 (range = 384.1) months (Fig. 1A, Tables 1, 2). Supplementary Table S1 describes the patients’ samples that are used per analysis (i.e. quantification and CNV profiling), the time point at which blood was withdrawn for either ctDNA quantification or CNV profiling, the known primary driver gene mutation, secondary driver gene mutation, BAP1 immunohistochemistry, and total follow-up per patient.

**Table 1.** Patient Characteristics of Fractionated Stereotactic Radiotherapy (fSRT) Cohort Used for Evaluation of Circulating Tumor DNA (ctDNA) Abundance, Which is Split Into Patients That had Detectable ctDNA (*n* = 3) and Patients With ctDNA Levels Under the Detection Limit (*n* = 3)

		fSRT Cohort		Disease State		
		Detectable ctDNA	Undetectable ctDNA	Localized	Metastatic	*P* Value
**Age at onset**	Median (range) in years	61 (20.8)	68.3 (59.3)	61.0 (59.3)	61.2 (43.3)	0.99
**Sex**	Male	2	1	6	6	0.87
	Female	1	2	7	7	
**MFS**	Median (range) in months	18.7 (18.2)^*^	28.5 (4.4)	19.9 (40.8)	15.8 (188.5)	0.73
**OS**	Median (range) in months	31.6 (16.5)	28.5 (4.4)	27.2 (41.0)	23.3 (41.4)	0.91
**Tumor location**	Choroid	3	1	10	10	0.28
	Ciliary body	0	2	3	3	
**T-class**	1	0	0	0	0	0.81
	2	2	1	5	3	
	3	1	2	7	8	
	4	0	0	1	2	
**LTD**	Mean (SD) in millimeters	12.7 (1.5)	11.7 (1.4)	13.9 (2.7)	15.6 (2.4)	0.11
**Thickness**	Mean (SD) in millimeters	8.3 (3.0)	8.7 (2.8)	7.3 (3.1)	7.8 (4.4)	0.92
**Ciliary body**	Yes	0	0	5	5	0.47
**Involvement**	No	0	1	3	3	
	NA	3	2	5	5	
**Extraocular spread**	Yes	0	0	2	3	0.62
	No	0	1	4	5	
	NA	3	2	7	5	
**Cytomorphology**	Epithelioid	1	1	3	2	**0.018**
	Mixed	1	0	6	6	
	Spindle	2	2	0	0	
	NA	0	0	4	5	
**Extracellular matrix patterns**	Yes	0	1	5	5	0.73
	No	0	0	1	2	
	NA	3	2	7	6	
**Mitotic count**	High (≥ 10/8 mm^2^)	0	0	1	2	0.58
	Low (< 10/8 mm^2^)	0	2	4	5	
	NA	3	1	8	6	
**Chromosome 3 loss**	Yes	1	2	7	9	0.50
	No	1	1	2	0	
	NA	1	0	4	4	
**Chromosome 6p gain**	Yes	1	0	3	2	0.73
	No	1	3	6	7	
	NA	1	0	4	4	
**Chromosome 8q gain**	Yes	1	2	6	6	0.96
	No	1	1	3	3	
	NA	1	0	4	4	
**Mutated primary driver gene**	*GNAQ* c.626A>C (Q209P)	2	0	4	6	0.77
	*GNAQ* c.626A>T (Q209L)	1	1	2	2	
	*GNA11* c.626A>T (Q209L)	0	2	6	4	
	*PLCB4* c.1888delinsTT (D630Y)	0	0	1	1	
**Mutated secondary driver gene**	*BAP1*	2	1	8	9	0.46
	*SF3B1*	0	0	1	1	
	*EIF1AX*	1	1	1	0	
	*MET*	0	1	1	0	
	NA	0	0	2	3	

NA, not applicable.

Additionally, ctDNA abundance was evaluated for patients with localized (at time of diagnosis; *n* = 13) and metastatic (*n* = 13) disease. Of these patients, eight patients had blood analyzed both in localized and metastatic disease. Histopathological and cytogenetic analyses were performed on tumor tissue obtained after biopsy or surgical removal of the tumor.

* One patient contracted metastases within the short timeframe of the study.

**Table 2.** Patient Characteristics of Patients Included for Shallow Whole Genome Sequencing (sWGS) With Localized and Metastatic Disease

		Localized *n* =17^*^	Metastatic *n* = 10^*^	*P* Value
**Age at onset**	Mean (range) in years	62.1 (40.7)	67.3 (43.4)	0.24
**Sex**	Male	5	4	0.68
	Female	12	6	
**Follow-up**	Median (range) in months	30.4 (384.1)	25.0 (78.4)	0.37
**Tumor location**	Choroid	14	10	0.27
	Ciliary body	3	0	
**T-class**	1	2	0	0.20
	2	7	2	
	3	8	8	
	4	0	0	
**Longest tumor diameter**	Mean (SD) in millimeters	12.3 (2.0)	14.9 (2.2)	**0.0045**
**Thickness**	Mean (SD) in millimeters	6.8 (2.9)	8.5 (4.5)	0.25
**Ciliary body involvement**	Yes	4	2	0.50
	No	2	3	
	NA	11	5	
**Extraocular spread**	Yes	0	2	0.12
	No	4	3	
**Cytomorphology**	NA	13	5	
	Epithelioid	3	2	0.38
	Mixed	3	4	
	Spindle	0	0	
	NA	11	4	
**Extracellular matrix patterns**	Yes	2	5	0.068
**Cytomorphology**	No	2	0	
	NA	13	5	
**Mitotic count**	High (≥ 10/8 mm^2^)	1	3	0.18
	Low (< 10/8 mm^2^)	3	2	
**Chromosome 3 loss**	NA	14	5	
	Yes	7	6	0.44
	No	0	0	
	NA	10	4	
**Chromosome 6p gain**	Yes	1	0	0.60
	No	6	5	
	NA	10	5	
**Chromosome 8q gain**	Yes	6	4	0.45
	No	1	2	
	NA	10	4	
**Primary driver mutation**	*GNAQ* c.626A>C (Q209P)	3	3	0.76
	*GNAQ* c.626A>T (Q209L)	2	1	
	*GNA11* c.626A>T (Q209L)	2	2	
	NA	10	4	
**Secondary driver**	*BAP1*	6	5	0.26
**mutation** ^†^	*SF3B1*	0	1	
	NA	11	4	

NA, not applicable.

* One patient had blood analyzed at diagnosis and at the detection of metastases (see Fig. 1A, Supplementary Table S1).

† One patient had an SF3B1 and BAP1 mutated tumor, with partially lost BAP1 expression.

### ctDNA Detection in Localized and Metastatic Disease

The ctDNA levels from peripheral blood were assessed in 13 patients in localized disease, prior to treatment and 13 patients at the detection of metastases (see Fig. 1A, Table 1). In localized disease, no patient displayed signs of metastases. Patients with primary UM had a median ctDNA abundancy of 0 copies per mL (range = 11.2), and 5 of 13 (38%) patients had an abundancy of ctDNA fragments above the detection limit. At the detection of metastases, more ctDNA copies per mL were detected compared to localized disease (median 24 copies per mL, range = 9000 copies/mL, *P* < 0.001) and 10 of the 13 (77%) patients had detectable ctDNA (Fig. 1B). We compared clinical and histopathological parameters of the tumors in localized disease with ctDNA abundancy. Five patients had an American Joint Commission on Cancer (AJCC) T-stage of 2, 7 patients had an AJCC T-stage of 3, and one had an AJCC T-stage of 4 (median ctDNA levels = 3, 0, and 3.5, respectively). Ten patients had a tumor originating in the choroid and three had a tumor in the ciliary body (median ctDNA levels = 3.1 and 0, respectively). BAP1 protein expression was known for 12 patients of which 8 had a BAP1 negative tumor and 4 had a BAP1 positive tumor (mean ctDNA levels = 4.2 and 3.2, respectively; see Table 1). The ctDNA abundancy was similar between these histopathological groups (*P* > 0.05; Figs. 1C–E).

### ctDNA Detection in Patients Undergoing fSRT

Nineteen patients underwent fSRT between December 12, 2019, and June 1, 2022, and had blood prospectively collected during fSRT. The primary driver mutation needed for ctDNA detection was known for 6 of 19 patients. Of these six patients, four patients underwent an endoresection, one patient underwent an enucleation, and one patient contracted metastases that was biopsied of which tumor material was analyzed. These patients had a median age at onset of 64.7 (range = 59.3) years and a median metastatic free survival of 19.2 months at the moment of data collection in this study. One patient developed metastases within the timeframe of this study (after 7.2 months). The tumors had a T-class of 2 (*n* = 3) and 3 (*n* = 3; see Table 1). Levels of ctDNA were quantified for six patients during fSRT. In three of six patients, ctDNA fragments were detected by dPCR prior to irradiation. In these patients, we also detected ctDNA fragments throughout fSRT. The ctDNA fragments were below the threshold of detection during the treatment in patients without detectable ctDNA fragments prior to fSRT. Levels of ctDNA copies did not change significantly in the patients with detectable ctDNA on the different days after the start of fSRT (Fig. 1F).

### CNV Profiling from Peripheral Blood

To determine the efficacy of sWGS for minimally invasive diagnostics and prognostication in patients with UM, we have performed sWGS on blood of 26 patients (see Table 2). These patients had blood available for sWGS and, if applicable, ctDNA levels were above zero. In this pilot study, blood samples were obtained for sWGS from nine patients with localized disease, prior to treatment. During fSRT, blood samples were collected from five patients. Blood samples were obtained from four patients during the follow-up period after treatment of the primary tumor, but prior to the detection of metastases (follow-up range = 3–387 months). At the time of metastasis detection, blood samples were collected from six patients. Finally, during the follow-up of metastatic disease, blood samples were obtained from four patients (see Fig. 1A).

**Figure 2.**
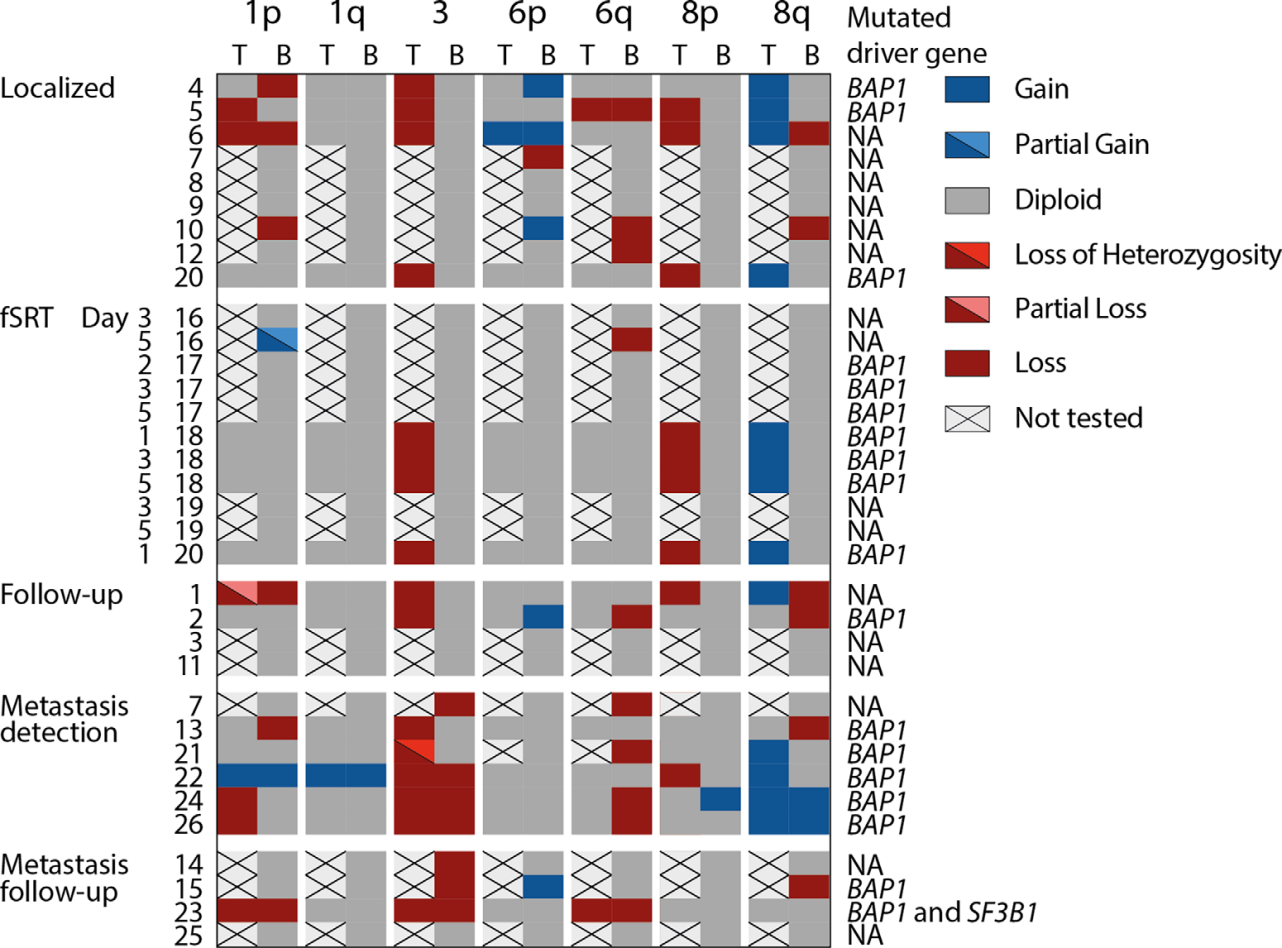
Comparison of detected CNVs in the tumor (when available) and in blood. Blood of nine patients was shallow whole genome sequenced (sWGS) in localized disease, prior to therapy. Five patients had blood analyzed during fractionated stereotactic radiotherapy (fSRT). Four patients had blood analyzed during follow-up after treatment of the primary tumor. Six patients had blood analyzed at the detection of metastases. Four patients had blood analyzed during follow-up of metastatic disease. Patient no. 20 had blood withdrawn at localized and fSRT time points and no informative CNV profile was obtained. Patient no. 7 had blood withdrawn at diagnosis and at detection of metastases and chromosome 3 loss (associated with high metastatic risk) was detected in plasma retrieved at the detection of metastases, whereas sWGS from plasma retrieved at diagnosis did not reveal CNVs associated with high metastatic risk. Chromosome 3 loss was detected in 7 of 10 patients with metastatic disease, of which patient no. 21 had loss of heterozygosity of chromosome 3 in the tumor. Patient and tumor characteristics (like primary and secondary driver mutation) are described in Supplementary Table S1. Abbreviations: T, tumor; B, blood; fSRT, fractionated stereotactic radiotherapy.

**Figure 3.**
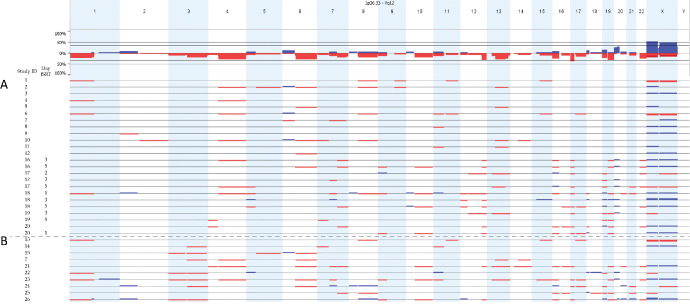
Summary of CNV profiles derived from plasma of patients with localized (*n* = 29) (**A**) and metastatic (*n* = 10) (**B**) uveal melanoma (UM). Chromosome 3 loss is detected in 7 of 10 patients harboring metastatic disease. In localized disease, no chromosome 3 loss was detected, even though chromosome 3 is lost in the primary tumor in several patients (see Fig. 2). The samples 1 to 15 were sequenced with the NIPT version 1 and samples 16 to 26 were sequenced with Veriseq-NIPT Solution version 2.

**Figure 4.**
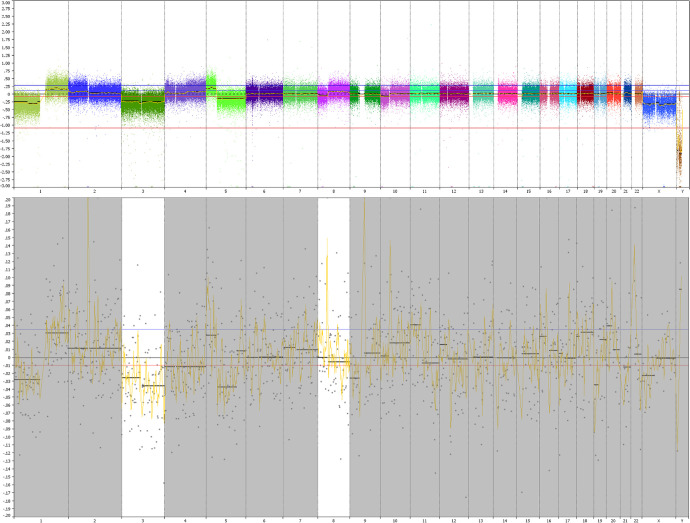
Corresponding CNV-profiles in patient harboring metastases. This patient developed several diffuse metastatic lesions in the liver, spleen, and spine. (**A**) Copy number variations of the primary tumor were acquired by single nucleotide polymorphism array of tumor tissue obtained after enucleation. The primary tumor shows a loss of chromosomes 1p, 3, 5q, and 10p; and a gain of chromosomes 1q and 8q. Tumor tissue is processed by the pathology laboratory within 4 hours after removal of tissue and subsequently molecular analyses is performed. In addition to routine diagnostics, peripheral blood was obtained after detection of metastases ultrasound of the liver that was later confirmed by computed tomography imaging. After in silico size selection, the loss of chromosomes 1p, 3, 8, and 10 and the gain of chromosome 1q were confirmed in the blood of this patient.

We optimized the detection of chromosomal aberrations for detecting chromosome 3 loss. We did not obtain CNV profiles corresponding to the primary tumor in blood of patients with localized disease, during follow-up, or during fSRT (Figs. 2, 3A). Interestingly, we confirmed the loss of chromosome 3 in 7 of 10 (70%) patients harboring metastatic disease (see Figs. 2–4). One patient had a loss of heterozygosity of chromosome 3 and, as we detect CNV profiles based on copy number, this allelic imbalance was not detected using sWGS on peripheral blood (see Fig. 2).

### Evaluation of ctDNA Quantity Needed for CNV Profiling

The sWGS requires a minimum input of aberrant (or tumor) DNA for the detection of chromosomal aberrations in the total amount of cfDNA. Guided by the data regarding our patients with UM blood samples, we noticed that the tumor load in patients harboring metastatic disease is, generally, sufficient for CNV detection by sWGS. We could obtain reliable CNV-profiles in patients that contracted metastases and in metastatic disease more ctDNA copies per mL are detected (see Fig. 1B).

To elucidate the amount of tumor derived circulating DNA needed for detection with sWGS, we performed in silico dilutions of chromosome 3 loss and chromosome 8q gain. The loss of chromosome 3 and gain of chromosome 8q was detectable from an in silico spiked in fraction of 1% and 1%, respectively (Supplementary Fig. S2).

## Discussion

In this pilot study, we assessed the feasibility of using ctDNA as a biomarker for disease progression and tumor characterization. We included 34 patients in a 5-year time period. Out of these, we included 6 of 19 patients who underwent fSRT, primarily due to the unavailability of tumor tissue in this subset of patients. Unfortunately, the sample size is limited (*n* = 34), especially for patients undergoing fSRT (*n* = 6) and more patients should be included to draw definite conclusions. We observed a rise in ctDNA levels in patients harboring metastatic UM compared to localized disease and this could serve as a biomarker for disease progression. During the 5 consecutive days of fSRT, ctDNA abundancy levels did not change. Detection of chromosome 3 loss from ctDNA is feasible when ctDNA abundancy is sufficient, which is typically observed in metastatic disease.

In primary UM, the tumor is small, which is resembled by the tumor load detected in blood. Our results corroborate the finding that in patients harboring metastatic UM, ctDNA is detected more often and in higher quantities. Previous studies focused on detecting and quantifying ctDNA using ultradeep sequencing and bidirectional pyrophosphorolysis-activated polymerization (Bi-PAP).^35^^,^^36^ Afterward, the sensitive droplet dPCR technique became more prevalent for establishing an easier detection of metastases and higher ctDNA levels correlated to disease progression.^21^^,^^24^^,^^37^ The most commonly published droplet dPCR method describes using the Qiagen circulating nucleic acid isolation kit on plasma derived from either EDTA or nucleic acid preserving blood collection tubes, after which the Bio-RAD assay is used. Results of obtaining ctDNA fragments above the detection limit vary from 26%,^24^ 29%,^38^ up to 100%.^39^ Bustamante et al.^39^ detected ctDNA in all 14 of 14 patients with primary UM. In this study, we used a similar approach and detected ctDNA in 5 of 13 (38%) patients with localized disease, prior to treatment, and in 10 of 13 (77%) patients with metastatic disease. Due to the availability of plasma, not every sample contained similar quantities of plasma. This could potentially be a factor reducing detection rate. However, we have not observed a difference in detection of non-mutated cfDNA between samples. In animal models, ctDNA levels correlate to tumor size and a rise in ctDNA levels preceded clinical manifestation of metastases.^39^ Next to detection of metastases, ctDNA abundance can be used for assessing minimal residual disease. Therapeutic studies on metastatic UM evaluate the efficacy for tebentafusp and pembrolizumab and entinostat by assessing ctDNA levels.^40^^,^^41^ Other studies have focused on the evaluation of protein kinase C inhibitors^18^ and anti-PD1 antibody therapies.^42^ In aqueous humor, ctDNA is detected and CNV profiles concordant to that of the tumor are found.^43^ However, this still requires an invasive biopsy of the anterior chamber.

Due to the infrequent detection of ctDNA in patients with localized disease, there was a need to enhance this for CNV profiling purposes. To address this issue, we explored ways to increase the limited amount of ctDNA that is observed in localized disease. We measured ctDNA levels during radiation treatment, as, theoretically, an irradiated tumor would shed ctDNA into the circulation.^38^^,^^44^^,^^45^ For example, ctDNA levels were undetectable in rabbits inoculated with tumor cells, but these ctDNA levels had risen during radiotherapy to a detectable level and showed a rapid decline after the therapy had ended.^44^ DNA from Epstein-Barr virus particles released from tumor cells in plasma was higher immediately after radiation, consistent with release due to therapy induced tumor cell death.^45^ During brachytherapy, ctDNA was significantly more detected in patients 2 days after applying the ionizing plaque.^38^ We have analyzed ctDNA abundancy during fSRT and the preliminary results show no change in abundancy during fSRT. This could be due to the low sample numbers or due to a biological effect. Suesskind et al.^46^ showed that radiation treatment does not elevate the number of biomarkers shed in blood. Another study in which three of five patients (60%) did not yield detectable ctDNA levels at baseline and ctDNA levels did not increase during or after radiation therapy for rectum carcinoma.^47^ One reason could be a delayed tumor cell death, as radiation therapy induces DNA damage by single base damage, single strand breaks, and double strand breaks and stop replication and transcription, resulting in cell death.^48^ Additionally, radiation therapy was shown to induce cell cycle arrest for up to 5 days in uveal melanoma cell line cells (92.1).^49^ Radiation induces an immune response by the pro-inflammatory signals that the stressed cells send out, which attracts, among others, T-lymphocytes and natural killer (NK)-cells.^50^ Additionally, tumor cells that died non-immunogenically are cleared by macrophages.^51^ The combined effect of delayed cell death and clearance by macrophages could lead to lower ctDNA levels than expected after radiation therapy. Furthermore, the sequence needed for digital PCR could be altered by double-strand breaks and crosslinks due to the irradiation, hampering the detection of tumor-derived DNA sequences.^48^

To assess CNV profiles from the tumor, NIPT was performed with the NIPT version 1 and the Veriseq NIPT Solution version 2, as this could be easily implemented clinically for the prognostication of patients with UM. During this study the workflow changed from NIPT version 1 to the Veriseq NIPT Solution version 2. The NIPT version 1 had a coverage of 0.2X and 51 bp single-end reads and the Veriseq NIPT Solution version 2 had a similar coverage and 36 bp paired-end reads. Regardless of the NIPT method used, chromosome 3 status could be confirmed in patients harboring metastatic UM.

Veriseq-NIPT was initially designed to detect fetal CNVs in maternal blood, and thus it is optimized to detect low levels of aberrated DNA within the overall maternal fraction. The NIPT analysis focuses on circulating cell-free fetal DNA, with a minimum fraction of 4% required for evaluation. In a study, it was found that 62% of fetal chromosomal aberrations were identified when the fetal fraction accounted for 4% of the total blood DNA. However, the detection rate improved to 100% when the fetal fraction reached 9%.^52^ NIPT is usually performed from 10 weeks of gestation when a fetus is around 30 millimeters long until 22 weeks of gestation when the fetus is 28 centimeters long. Between that period the fetal fraction of cfDNA remains similar.^53^ In the Trident-2 study, Dutch pregnant women tested their blood for chromosomal aberrations of the fetus. In 0.03% of the sWGS, a malignancy is discovered after chromosomal aberrations are observed. These cases primarily comprise hematological malignancies.^54^ Several studies reported on the detection of malignancies using NIPT sWGS. Cohen et al. detected 40% of high-grade ovarian carcinomas using a similar pipeline as our first run (0.2X, 36 bp, and single-end sequencing). In which they obtained CNV profiles of 6 of the 16 patients with early stage and 7 of the 16 late stage ovarian carcinomas.^55^ Most studies that report on successfully obtained ctDNA derived CNV profiles show a ctDNA fraction of more than 10%.^33^^,^^56^^,^^57^ Furthermore, the detection limit of IchorCNA to detect and evaluate tumor fraction is 3% ctDNA in cfDNA using sWGS.^33^^,^^57^ In primary UM, a threshold of 3% tumor fraction is not feasible as the tumor is too small and ctDNA copies are sparse. In non-small cell lung cancer (NSCLC), the tumor volume is correlated to mean tumor fraction recovered from plasma. From this correlation, they predicted that a tumor with a volume of 1 cm^3^ corresponds to a tumor fraction of 0.008% and a tumor with a volume of 10 cm^3^ corresponds to a tumor fraction of 0.1%.^58^ Calculating tumor volume for primary UMs is difficult as these tumors have a mushroom, dome, or asymmetric shape. Considering these tumor shapes, one study estimated tumor volumes of UMs in two cohorts comprised of patients with Gaussian distributed AJCC T-stages and tumor-thickness classes (i.e. most tumors had a T-class of 2 or 3 and a thickness of 9 mm) and about two thirds of the patients with UM had a lower volume than 1 cm^3^.^59^ When the ctDNA tumor fraction is extrapolated from NSCLC to the tumor size of primary UM, the tumor fraction is less than 0.1% and insufficient for deriving CNV profiles, as is shown with CNVs in fetal fractions.^52^ Therefore, we selected DNA fragments of 80 to 150 bp in the paired-end sWGS samples. Previous studies report on the added benefit of size selection,^30^^,^^31^ as ctDNA strands are reported to be smaller than regular cfDNA strands. The cfDNA strands comprise the histone with linker DNA and are mostly shed through apoptosis.^60^ The ctDNA fragment sizes are more diverse, as is expected by its irregular fragmentation.^60^ Additionally, fetal circulating DNA is shorter than maternal cfDNA,^61^ providing the opportunity to incorporate in silico size-selection into the fetal NIPT testing and sequencing short cfDNA fragments improved fetal fraction.^62^

We have shown that loss of chromosome 3 could be detected in peripheral blood of patients harboring metastatic UM, as they have adequate ctDNA levels. Several therapeutic studies are conducted to target metastasized UM cells. Tebentafusp is known to activate the immune system toward UM cells and shows longer overall survival compared to other immunomodulating therapies.^63^ Tebentafusp can be universally used in patients with UM as it is targeted to glycoprotein 100 (gp100) but requires a specific HLA subtype (HLA-A*02:01). Other studies target the secondary driver mutations responsible for metastasizing.^64^ As *SF3B1* mutations affect splicing, different therapeutics are tested to attack cells with abnormal splicing activity.^65^^,^^66^ These *BAP1* and *SF3B1* mutated tumors have distinct CNV-profiles and, in our pilot study, we have shown that chromosome 3 loss can be identified in patients harboring metastases. Therefore, sWGS on plasma-derived ctDNA could be used for the selection of patients in trials targeting *BAP1* or *SF3B1* mutated tumors and metastases.

## Supplementary Material

Supplement 1

## References

[1] de Bruyn DP, Beasley AB, Verdijk RM, et al. Is tissue still the issue? The promise of liquid biopsy in uveal melanoma. *Biomedicines*. 2022; 10: 506. 35203714 10.3390/biomedicines10020506 PMC8962331

[2] Klingenstein A, Fürweger C, Mühlhofer AK, et al. Quality of life in the follow-up of uveal melanoma patients after enucleation in comparison to CyberKnife treatment. *Graefe's Arch Clinic Exp Ophthalmol*. 2016; 254: 1005–1012. 10.1007/s00417-015-3216-7 26573389

[3] Frizziero L, Midena E, Trainiti S, et al. Uveal melanoma biopsy: a review. *Cancers*. 2019; 11: 1075. 31366043 10.3390/cancers11081075 PMC6721328

[4] Bagger M, Tebering JF, Kiilgaard JF. The ocular consequences and applicability of minimally invasive 25-gauge transvitreal retinochoroidal biopsy. *Ophthalmology*. 2013; 120: 2565–2572. 24053996 10.1016/j.ophtha.2013.07.043

[5] Bechrakis NE, Foerster MH, Bornfeld N. Biopsy in indeterminate intraocular tumors. *Ophthalmology*. 2002; 109: 235–242. 11825801 10.1016/s0161-6420(01)00931-9

[6] Singh AD, Medina CA, Singh N, Aronow ME, Biscotti CV, Triozzi PL. Fine-needle aspiration biopsy of uveal melanoma: outcomes and complications. *Br J Ophthalmol*. 2016; 100: 456–462. 26231747 10.1136/bjophthalmol-2015-306921

[7] Cohen V, Dinakaran S, Parsons M, Rennie I. Transvitreal fine needle aspiration biopsy: the influence of intraocular lesion size on diagnostic biopsy result. *Eye*. 2001; 15: 143–147. 11339578 10.1038/eye.2001.48

[8] Faulkner-Jones BE, Wilham J. Fine needle aspiration biopsy with adjunct immunohistochemistry in intraocular tumor management. *Acta Cytologica*. 2005; 49: 297–308. 15966293 10.1159/000326153

[9] Jager MJ, Shields CL, Cebulla CM, et al. Uveal melanoma. *Nat Rev Dis Primers*. 2020; 6: 1–25. 32273508 10.1038/s41572-020-0158-0

[10] Robertson AG, Shih J, Yau C, et al. Integrative analysis identifies four molecular and clinical subsets in uveal melanoma. *Cancer Cell*. 2017; 32(2): 204–220.e15. 28810145 10.1016/j.ccell.2017.07.003 PMC5619925

[11] Yavuzyigitoglu S, Koopmans AE, Verdijk RM, et al. Uveal melanomas with SF3B1 mutations: a distinct subclass associated with late-onset metastases. *Ophthalmology*. 2016; 123: 1118–1128. 26923342 10.1016/j.ophtha.2016.01.023

[12] Drabarek W, Yavuzyigitoglu S, Obulkasim A, et al. Multi-modality analysis improves survival prediction in enucleated uveal melanoma patients. *Invest Ophthalmol Vis Sci*. 2019; 60: 3595–3605. 31425584 10.1167/iovs.18-24818

[13] Smit KN, Jager MJ, de Klein A, Kiliҫ E. Uveal melanoma: towards a molecular understanding. *Prog Retin Eye Res**.* 2020; 75: 100800. 31563544 10.1016/j.preteyeres.2019.100800

[14] Koopmans AE, Vaarwater J, Paridaens D, Naus NC, Kilic E, de Klein A. Patient survival in uveal melanoma is not affected by oncogenic mutations in GNAQ and GNA11. *Br J Cancer*. 2013; 109: 493–496. 23778528 10.1038/bjc.2013.299 PMC3721402

[15] Karlsson J, Nilsson LM, Mitra S, et al. Molecular profiling of driver events in metastatic uveal melanoma. *Nat Commun*. 2020; 11: 1894. 32313009 10.1038/s41467-020-15606-0 PMC7171146

[16] Shain AH, Bagger MM, Yu R, et al. The genetic evolution of metastatic uveal melanoma. *Nat Genet*. 2019; 51: 1123–1130. 31253977 10.1038/s41588-019-0440-9 PMC6632071

[17] Staby KM, Gravdal K, Mørk SJ, Heegaard S, Vintermyr OK, Krohn J. Prognostic impact of chromosomal aberrations and GNAQ, GNA11 and BAP1 mutations in uveal melanoma. *Acta Ophthalmol*. 2018; 96: 31–38. 28444874 10.1111/aos.13452

[18] Park JJ, Diefenbach RJ, Byrne N, et al. Circulating tumor DNA reflects uveal melanoma responses to protein kinase C inhibition. *Cancers*. 2021; 13: 1740. 33917514 10.3390/cancers13071740 PMC8038771

[19] McEvoy AC, Pereira MR, Reid A, et al. Monitoring melanoma recurrence with circulating tumor DNA: a proof of concept from three case studies. *Oncotarget*. 2019; 10: 113–122. 30719207 10.18632/oncotarget.26451 PMC6349444

[20] Palmieri M, Baldassarri M, Fava F, et al. Two-point-NGS analysis of cancer genes in cell-free DNA of metastatic cancer patients. *Cancer Med*. 2020; 9: 2052–2061. 31991072 10.1002/cam4.2782 PMC7064095

[21] Le Guin CH, Bornfeld N, Bechrakis NE, et al. Early detection of metastatic uveal melanoma by the analysis of tumor-specific mutations in cell-free plasma DNA. *Cancer Med**.* 2021; 10: 5974–5982. 34291585 10.1002/cam4.4153 PMC8419753

[22] Rostami A, Lambie M, Yu CW, Stambolic V, Waldron JN, Bratman SV. Senescence, necrosis, and apoptosis govern circulating cell-free DNA release kinetics. *Cell Rep*. 2020; 31: 107830. 32610131 10.1016/j.celrep.2020.107830

[23] Kotecha R, Tonse R, Menendez MAR, et al. Evaluation of the impact of pre-operative stereotactic radiotherapy on the acute changes in histopathologic and immune marker profiles of brain metastases. *Sci Rep*. 2022; 12: 4567. 35296750 10.1038/s41598-022-08507-3 PMC8927473

[24] Beasley A, Isaacs T, Khattak MA, et al. Clinical application of circulating tumor cells and circulating tumor DNA in uveal melanoma. *JCO Precis Oncol*. 2018; 2(2): 1–12. 10.1200/PO.17.00279 PMC7446501 32913999

[25] van Prooyen Schuurman L, Sistermans EA, Van Opstal D, et al. Clinical impact of additional findings detected by genome-wide non-invasive prenatal testing: Follow-up results of the TRIDENT-2 study. *Am J Hum Genet*. 2022; 109: 1140–1152. 35659929 10.1016/j.ajhg.2022.04.018 PMC9247828

[26] Li H, Durbin R. Fast and accurate short read alignment with Burrows-Wheeler transform. *Bioinformatics*. 2009; 25: 1754–1760. 19451168 10.1093/bioinformatics/btp324 PMC2705234

[27] Broadinstitute. Available at: http://broadinstitute.github.io/picard/.

[28] Koopmans AE, Verdijk RM, Brouwer RWW, et al. Clinical significance of immunohistochemistry for detection of BAP1 mutations in uveal melanoma. *Modern Pathology*. 2014; 27: 1321–1330. 24633195 10.1038/modpathol.2014.43

[29] Smit KN, van Poppelen NM, Vaarwater J, et al. Combined mutation and copy-number variation detection by targeted next-generation sequencing in uveal melanoma. *Mod Pathol*. 2018; 31: 763–771. 29327717 10.1038/modpathol.2017.187

[30] Mouliere F, Chandrananda D, Piskorz AM, et al. Enhanced detection of circulating tumor DNA by fragment size analysis. *Sci Transl Med**.* 2018; 10: eaat492. 10.1126/scitranslmed.aat4921 PMC6483061 30404863

[31] Peneder P, Stütz AM, Surdez D, et al. Multimodal analysis of cell-free DNA whole-genome sequencing for pediatric cancers with low mutational burden. *Nat Commun*. 2021; 12: 3230. 34050156 10.1038/s41467-021-23445-w PMC8163828

[32] Danecek P, Bonfield JK, Liddle J, et al. Twelve years of SAMtools and BCFtools. *Gigascience*. 2021; 10: giab008. 33590861 10.1093/gigascience/giab008 PMC7931819

[33] Adalsteinsson VA, Ha G, Freeman SS, et al. Scalable whole-exome sequencing of cell-free DNA reveals high concordance with metastatic tumors. *Nat Communic*. 2017; 8: 1–13. 10.1038/s41467-017-00965-y PMC5673918 29109393

[34] Quinlan AR, Hall IM. BEDTools: a flexible suite of utilities for comparing genomic features. *Bioinformatics*. 2010; 26: 841–842. 20110278 10.1093/bioinformatics/btq033 PMC2832824

[35] Metz CHD, Scheulen M, Bornfeld N. Ultradeep sequencing detects GNAQ and GNA11 mutations in cell-free DNA from plasma of patients with uveal melanoma. *Cancer Med*. 2013; 2(2): 208–215. 23634288 10.1002/cam4.61 PMC3639659

[36] Madic J, Piperno-Neumann S, Servois V, et al. Pyrophosphorolysis-activated polymerization detects circulating tumor DNA in metastatic uveal melanoma. *Clin Cancer Res*. 2012; 18: 3934–3941. 22645051 10.1158/1078-0432.CCR-12-0309

[37] Bidard FC, Madic J, Mariani P, et al. Detection rate and prognostic value of circulating tumor cells and circulating tumor DNA in metastatic uveal melanoma. *Int J Cancer*. 2014; 134: 1207–1213. 23934701 10.1002/ijc.28436

[38] Francis JH, Barker CA, Brannon AR, et al. Detectability of plasma-derived circulating tumor DNA panel in patients undergoing primary treatment for uveal melanoma. *Invest Ophthalmol Vis Sci*. 2022; 63: 17. 10.1167/iovs.63.13.17 PMC9766787 36525262

[39] Bustamante P, Tsering T, Coblentz J, et al. Circulating tumor DNA tracking through driver mutations as a liquid biopsy-based biomarker for uveal melanoma. *J Exp Clinic Cancer Res*. 2021; 40: 1–16. 10.1186/s13046-021-01984-w PMC8207750 34134723

[40] Carvajal RD, Butler MO, Shoushtari AN, et al. Clinical and molecular response to tebentafusp in previously treated patients with metastatic uveal melanoma: a phase 2 trial. *Nat Med*. 2022; 28: 2364–2373. 36229663 10.1038/s41591-022-02015-7 PMC9671803

[41] Ny L, Jespersen H, Karlsson J, et al. The PEMDAC phase 2 study of pembrolizumab and entinostat in patients with metastatic uveal melanoma. *Nat Commun*. 2021; 12: 5155. 34453044 10.1038/s41467-021-25332-w PMC8397717

[42] Cabel L, Riva F, Servois V, et al. Circulating tumor DNA changes for early monitoring of anti-PD1 immunotherapy: a proof-of-concept study. *Ann Oncol*. 2017; 28: 1996–2001. 28459943 10.1093/annonc/mdx212

[43] Im DH, Peng CC, Xu L, et al. Potential of aqueous humor as a liquid biopsy for uveal melanoma. *Int J Mol Sci*. 2022; 23(11): 6226. 35682905 10.3390/ijms23116226 PMC9181140

[44] Muhanna N, Eu D, Chan HHL, et al. Cell-free DNA and circulating tumor cell kinetics in a pre-clinical head and neck cancer model undergoing radiation therapy. *BMC Cancer*. 2021; 21: 1075. 34600526 10.1186/s12885-021-08791-8 PMC8487588

[45] Lo YM, Leung SF, Chan LY, et al. Kinetics of plasma Epstein-Barr virus DNA during radiation therapy for nasopharyngeal carcinoma. *Cancer Res*. 2000; 60: 2351–2355. 10811107

[46] Suesskind D, Ulmer A, Schiebel U, et al. Circulating melanoma cells in peripheral blood of patients with uveal melanoma before and after different therapies and association with prognostic parameters: a pilot study. *Acta Ophthalmol*. 2011; 89: 17–24. 21272286 10.1111/j.1755-3768.2009.01617.x

[47] Kuligina E, Moiseyenko F, Belukhin S, et al. Tumor irradiation may facilitate the detection of tumor-specific mutations in plasma. *World J Clin Oncol*. 2021; 12: 1215–1226. 35070740 10.5306/wjco.v12.i12.1215 PMC8716992

[48] Nakano T, Mitsusada Y, Salem AM, et al. Induction of DNA-protein cross-links by ionizing radiation and their elimination from the genome. *Mutat Res*. 2015; 771: 45–50. 25771979 10.1016/j.mrfmmm.2014.12.003

[49] He J, Li J, Ye C, et al. Cell cycle suspension: a novel process lurking in G₂ arrest. *Cell Cycle*. 2011; 10: 1468–1476. 21455017 10.4161/cc.10.9.15510

[50] Kaminski JM, Shinohara E, Summers JB, Niermann KJ, Morimoto A, Brousal J. The controversial abscopal effect. *Cancer Treat Rev*. 2005; 31: 159–172. 15923088 10.1016/j.ctrv.2005.03.004

[51] de Andrade Carvalho H, Villar RC. Radiotherapy and immune response: the systemic effects of a local treatment. *Clinics*. 2018; 73: e557s. 30540123 10.6061/clinics/2018/e557s PMC6257057

[52] Wright D, Wright A, Nicolaides KH. A unified approach to risk assessment for fetal aneuploidies. *Ultrasound Obstet Gynecol*. 2015; 45: 48–54. 25315809 10.1002/uog.14694

[53] Norton ME, Brar H, Weiss J, et al. Non-Invasive Chromosomal Evaluation (NICE) study: results of a multicenter prospective cohort study for detection of fetal trisomy 21 and trisomy 18. *Am J Obstet Gynecol*. 2012; 207: 137.e131–137.e138. 10.1016/j.ajog.2012.05.021 22742782

[54] Heesterbeek CJ, Aukema SM, Galjaard R-JH, et al. Noninvasive prenatal test results indicative of maternal malignancies: a nationwide genetic and clinical follow-up study. *J Clin Oncol*. 2022; 40: 2426–2435. 35394817 10.1200/JCO.21.02260

[55] Cohen PA, Flowers N, Tong S, Hannan N, Pertile MD, Hui L. Abnormal plasma DNA profiles in early ovarian cancer using a non-invasive prenatal testing platform: implications for cancer screening. *BMC Med*. 2016; 14: 126. 27558279 10.1186/s12916-016-0667-6 PMC4997750

[56] Wei T, Zhang J, Li J, et al. Genome-wide profiling of circulating tumor DNA depicts landscape of copy number alterations in pancreatic cancer with liver metastasis. *Molec Oncol*. 2020; 14: 1966–1977. 32593194 10.1002/1878-0261.12757 PMC7463305

[57] Lenaerts L, Che H, Brison N, et al. Breast cancer detection and treatment monitoring using a noninvasive prenatal testing platform: utility in pregnant and nonpregnant populations. *Clinic Chem*. 2020; 66: 1414–1423. 10.1093/clinchem/hvaa196 33141904

[58] Abbosh C, Birkbak NJ, Wilson GA, et al. Phylogenetic ctDNA analysis depicts early-stage lung cancer evolution. *Nature*. 2017; 545: 446–451. 28445469 10.1038/nature22364 PMC5812436

[59] Arnljots TS, Al-Sharbaty Z, Lardner E, All-Eriksson C, Seregard S, Stålhammar G. Tumour thickness, diameter, area or volume? The prognostic significance of conventional versus digital image analysis-based size estimation methods in uveal melanoma. *Acta Ophthalmol*. 2018; 96: 510–518. 29338132 10.1111/aos.13668

[60] Mouliere F, Rosenfeld N. Circulating tumor-derived DNA is shorter than somatic DNA in plasma. *Proc Natl Acad Sci USA*. 2015; 112: 3178–3179. 25733911 10.1073/pnas.1501321112 PMC4371901

[61] Yu SC, Chan KC, Zheng YW, et al. Size-based molecular diagnostics using plasma DNA for noninvasive prenatal testing. *Proc Natl Acad Sci USA*. 2014; 111: 8583–8588. 24843150 10.1073/pnas.1406103111 PMC4060699

[62] Qiao L, Zhang Q, Liang Y, et al. Sequencing of short cfDNA fragments in NIPT improves fetal fraction with higher maternal BMI and early gestational age. *Am J Transl Res*. 2019; 11: 4450–4459. 31396348 PMC6684886

[63] Nathan P, Hassel JC, Rutkowski P, et al. Overall survival benefit with tebentafusp in metastatic uveal melanoma. *N Engl J Med**.* 2021; 385: 1196–1206. 34551229 10.1056/NEJMoa2103485

[64] Seedor RS, Orloff M, Sato T. Genetic landscape and emerging therapies in uveal melanoma. *Cancers*. 2021; 13: 5503. 34771666 10.3390/cancers13215503 PMC8582814

[65] North K, Benbarche S, Liu B, et al. Synthetic introns enable splicing factor mutation-dependent targeting of cancer cells. *Nat Biotechnol*. 2022; 40: 1103–1113. 35241838 10.1038/s41587-022-01224-2 PMC9288984

[66] Maguire SL, Leonidou A, Wai P, et al. SF3B1 mutations constitute a novel therapeutic target in breast cancer. *J Pathol*. 2015; 235: 571–580. 25424858 10.1002/path.4483 PMC4643177

