# Supplementary material for: Evaluation of Circulating Tumor DNA as a Liquid Biomarker in Uveal Melanoma
Source: Invest Ophthalmol Vis Sci. 2024 Feb 6;65(2):11. doi: 10.1167/iovs.65.2.11 (PMC10854420; doi:10.1167/iovs.65.2.11)
Supplement: Supplement 1 [file iovs-65-2-11_s001.pdf]

Evaluation of circulating tumor DNA as a liquid biomarker in uveal melanoma

Daniel P. de Bruyn[1,2,3], Natasha M. van Poppelen[1,2,3], Tom Brands[2], Susanne C. van den Boom[2], Ellis Eikelenboom[2], Anja Wagner[2], Monique M. van Veghel-Plandsoen[2], Geert Geeven[2], Berna Beverloo[2], Robert M. Verdijk[4,5], Nicole C. Naus[1,2], Mette M. Bagger[6, 7], Jens F. Kiilgaard[6], Annelies de Klein[2,3], Erwin Brosens[2,3†] & Emine Kiliç[1,3†,*].

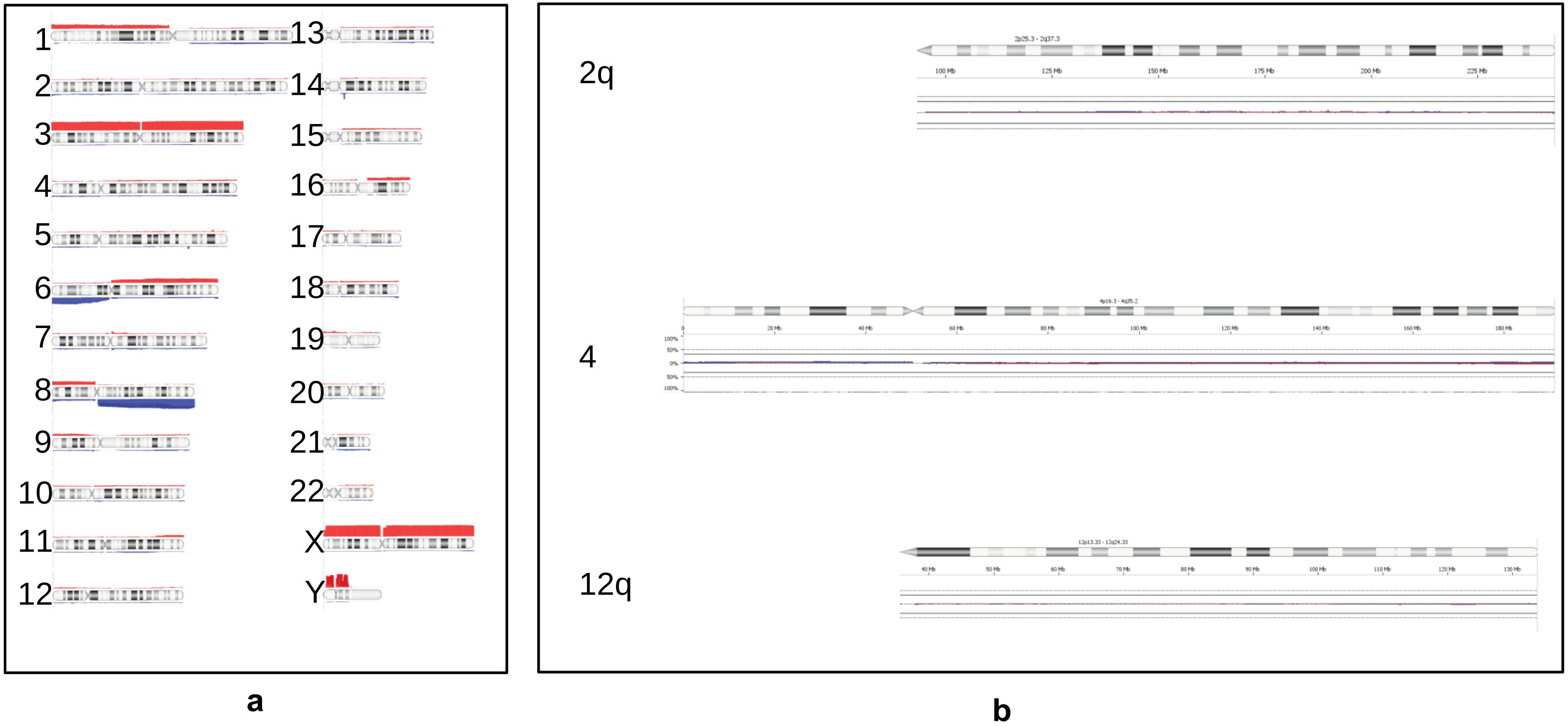

Supplementary figure 1. Distribution of chromosomal aberrations in Rotterdam Ocular Melanoma Study group (ROMS) uveal melanoma cohort. Uveal melanomas (UMs) are known to harbor few, typical, copy number variations. We have obtained 384 CNV-profiles of UMs by SNP-array. A loss is depicted in red above the respective chromosome and a gain is depicted in blue below the respective chromosome. In our cohort, a loss of chromosome 1, 3, 6q, and 8p and gain of chromosomes 6p and 8q are most frequently reported (**a**). The least frequently aberrated chromosome arms are 2q, 4 and 12q and have CNVs in less than five percent of the 384 UM-cases we evaluated by SNP-array (**b**).

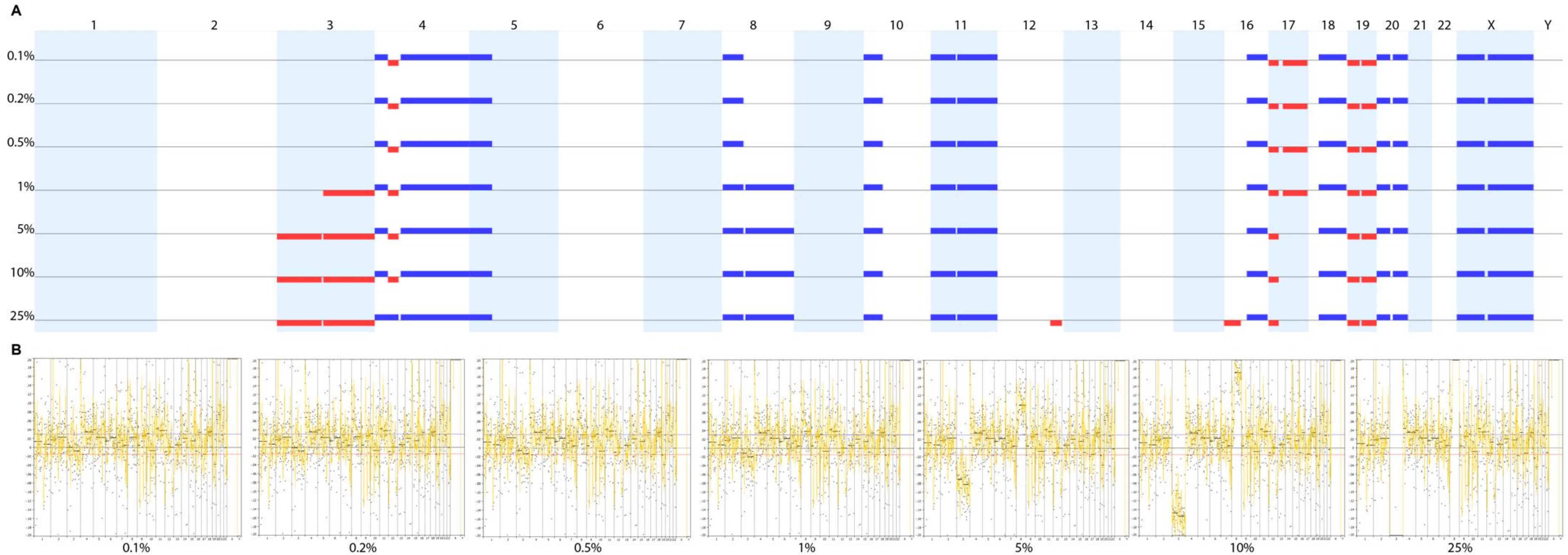

Supplementary figure 2. In silico spike in of chromosome 3 loss and chromosome 8q gain.
A concurrent loss of chromosome 3 and gain of chromosome 8q are correlated to the worst prognosis in UM. To derive a minimum fraction of tumor derived DNA in total cell free DNA, we made serial in-silico spike in dilutions of chromosome 3 loss and 8q gain. The dilutions show that with our pipeline we can detect 8q gain from 1% and chromosome 3 loss from 1%.

Supplementary table 1. Additional cohort description for patients included in this study. This includes the analyses patient samples were included in, the mutations found in the primary and secondary driver genes, BAP1 immunohistochemistry, and total follow-up. ctDNA was isolated to assess the abundancy (n=18) and to derive copy number variation (CNV) profiles (n=26). Ten patients had ctDNA analyzed for both assessing abundancy and CNV profiling. Tumor tissue was available for 22 of the 34 patients in this study. Abbreviations: fSRT: fractionated stereotactic radiotherapy; VUS: variant of unknown significance; CNV: copy number variation; IHC: immunohistochemistry; n.t.: not tested.

Study ID	Cohort	Subcohort	Time-point ctDNA abundancy	Time-point CNV profiling	Primary driver mutation	Secondary driver mutation	BAP1 IHC	Follow-up
	Abundancy	Diagnosis&fSRT	localized and fSRT	not applicable	GNAQ 626A>C Q209P	EIF1AX c.7A>G; p.K3E	Positive	18.7
	Abundancy	Diagnosis&fSRT	localized and fSRT	not applicable	GNA11 626A>T Q209L	(VUS) EIF1AX c.5C>T; p.P2L	Negative	27.2
	Abundancy	Diagnosis&fSRT	localized and fSRT	not applicable	GNAQ 626A>T Q209L	MET c.467C>T; p.S156L	Positive	28.5
	Abundancy	Diagnosis&metastasis	localized and metastatic	not applicable	PLCB4 D630. 1888delinsTT	SF3B1 c.1873C>T; R625C	Positive	21
	Abundancy	Diagnosis&metastasis	localized and metastatic	not applicable	GNA11 c.626A>T Q209L	n.t.	Negative	16.8
	Abundancy	Diagnosis&metastasis	localized and metastatic	not applicable	GNA11 c.626A>T Q209L	BAP1 c.2012-2026delinsCATCG	Negative	44.6
	Abundancy	Metastasis	metastatic	not applicable	GNAQ 626A>C Q209P	BAP1 (ex1): c.-3_15del	Negative	32.3
04	Abundancy&CNV	Diagnosis&metastasis&sWGS1	localized and metastatic	localized	GNAQ c.626A>C Q209P	BAP1 c.141_154del; p.I47Afs	Negative	50.1
06	Abundancy&CNV	Diagnosis&metastasis&sWGS1	localized and metastatic	localized	GNAQ c.626A>T Q209L	unknown	Positive	25.5
07	Abundancy&CNV	Diagnosis&metastasis&sWGS1	localized and metastatic	localized and metastatis detection	GNA11 c.626A>T (209L)	n.t.	n.t.	9.1
18	Abundancy&CNV	Diagnosis&fSRT&sWGS2	localized and fSRT	fSRT	GNA11 626A>T Q209L	unknown	Negative	31.6
20	Abundancy&CNV	Diagnosis&fSRT&sWGS2	localized and fSRT	localized and fSRT	GNAQ 626A>C Q209P	BAP1 c.947_954del; p.A316Vfs*79	Negative	31.6
17	Abundancy&CNV	Diagnosis&fSRT&metastasis&sWGS2	localized, fSRT, metastatic	fSRT	GNAQ: 626A>C Q209P	BAP1 c.1313_1319del; p.S438Cfs*131	Negative	35.2
22	Abundancy&CNV	Diagnosis&metastasis&sWGS2	localized and metastatic	metastatis detection	GNA11 c.626A>T Q209L	BAP1 c.1985_1988del; p.I662Mfs*29	Negative	16.7
23	Abundancy&CNV	Metastasis&sWGS2	metastatic	metastasic follow-up	GNAQ 626A>T Q209L	SF3B1 c.1873C>T; R625C and BAP1 c.1951_1955del; p.K651Gfs*3	Negative	11.0
24	Abundancy&CNV	Metastasis&sWGS2	metastatic	metastatis detection	GNAQ 626A>C Q209P	BAP1 (ex10): c.799_800del; p.Q267Afs*16	Negative	26.9
26	Abundancy&CNV	Metastasis&sWGS2	metastatic	metastatis detection	GNAQ 626A>C Q209P	BAP1 c.1671_1684del; p.S558Afs*4	Negative	8.7
01	CNV	sWGS1	not applicable	post treatment follow-up	GNAQ 626A>T Q209L	n.t.	Negative	11.3
02	CNV	sWGS1	not applicable	post treatment follow-up	n.t.	n.t.	Negative	153.2
03	CNV	sWGS1	not applicable	post treatment follow-up	n.t.	n.t.	n.t.	387
05	CNV	sWGS1	localized	localized	n.t.	n.t.	Negative	19.4
08	CNV	sWGS1	not applicable	localized	n.t.	n.t.	n.t.	4.9
09	CNV	sWGS1	not applicable	localized	n.t.	n.t.	n.t.	61.3
10	CNV	sWGS1	not applicable	localized	n.t.	n.t.	n.t.	56.9
11	CNV	sWGS1	not applicable	post treatment follow-up	n.t.	n.t.	n.t.	83.6
12	CNV	sWGS1	not applicable	localized	n.t.	n.t.	n.t.	2.9

13	CNV	sWGS1	not applicable	metastatis detection	GNAQ 626A>C Q209P	BAP1 c.302T>C; p.L101P	Negative	60.2
14	CNV	sWGS1	not applicable	metastatic follow-up	n.t.	n.t.	n.t.	25.1
15	CNV	sWGS1	not applicable	metastatic follow-up	GNAQ 626A>T Q209L	BAP1 c.1582_1583del; p.S528Ofs*8	Negative	51.6
16	CNV	sWGS2	not applicable	fSRT	n.t.	n.t.	n.t.	27.8
21	CNV	sWGS2	not applicable	metastatis detection	GNA11 626AG>TT Q209L	BAP1 c.1838del; p.T613Afs*4	Negative	87.1
19	CNV	sWGS2	not applicable	fSRT	n.t.	n.t.	n.t.	31.1
25	CNV	sWGS2	not applicable	metastatic follow-up	n.t.	n.t.	n.t.	24.9
	Abundancy	Metastasis	metastatic	not applicable	GNAQ 626A>C Q209P	unknown	n.t.	245.4

* Targeted sequencing did not reveal the driver mutation (*BAP1*, *SF3B1* and *EIF1AX*) in one patient
